# Organism‐Generated Biological Vesicles In Situ: An Emerging Drug Delivery Strategy

**DOI:** 10.1002/advs.202204178

**Published:** 2022-11-24

**Authors:** Li Kong, Conglian Yang, Zhiping Zhang

**Affiliations:** ^1^ Tongji School of Pharmacy Huazhong University of Science and Technology Wuhan 430030 P. R. China; ^2^ Hubei Engineering Research Center for Novel Drug Delivery System Huazhong University of Science and Technology Wuhan 430030 P. R. China; ^3^ National Engineering Research Center for Nanomedicine Huazhong University of Science and Technology Wuhan 430030 P. R. China

**Keywords:** bioinformation transfer, biological vesicles, disease treatment, drug delivery, in situ generation, organisms

## Abstract

Biological vesicles, containing genetic materials and proteins of the original cells, are usually used for local or systemic communications among cells. Currently, studies on biological vesicles as therapeutic strategies or drug delivery carriers mainly focus on exogenously generated biological vesicles. However, the limitations of yield and purity caused by the complex purification process still hinder their clinical transformation. Recently, it has been reported that living organisms, including cells and bacteria, can produce functional/therapeutic biological vesicles within body automatically. Therefore, using organisms to produce endogenous biological vesicles in body as drug/bio‐information delivery carriers has become a potential therapeutic strategy. In this review, the current development status and application prospects of in situ organism‐produced biological vesicles are introduced. The advantages and effects of this endogenous biological vesicles‐based strategy in drug delivery and disease treatments are analyzed. According to the type of endogenous biological vesicles, they are divided into four categories: exosomes, platelet‐derived microparticles, apoptotic bodies, and bacteria‐released outer membrane vesicles. And finally, the shortcomings of current research and future development are analyzed. This review is believed to open up the application of endogenous biological vesicles in the field of biomedicine and shed light on current research.

## Graphical abstract

In situ organism‐generated biological vesicles are a frontier field of drug delivery and disease treatment. Through transplant of modified organisms or in response to external stimuli, functional biological vesicles can be released in the body. This review presents a comprehensive summary of cutting‐edge development, advantages, and design strategies, and analyzes the perspectives and limitations of endogenous biological vesicles.

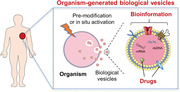

## 1 Introduction

Biological vesicles, including exosomes,^[^ ^1^ ^]^ outer membrane vesicles (OMVs) from bacteria,^[^ ^2^ ^]^ apoptotic bodies (ApoBDs), and microparticles,^[^ ^3^ ^]^ are nano/micro‐scale membrane vesicles secreted by organisms, cells, or bacteria. They usually contain genetic materials and proteins from the original cells and can be used for local or systemic communications among cells.^[^ ^4^ ^]^ The unique features of biological vesicles qualify them as an alternative therapeutic pathway and as available drug delivery systems to carry and protect drugs/nucleic acids to target sites. Therefore, many therapeutic strategies based on biological vesicles have been developed to treat diseases.^[^ ^5^ ^]^ Recent studies mainly focus on the use of exogenously generated biological vesicles as therapeutic strategies or drug delivery carriers. In other words, exogenous biological vesicles are obtained through cell culture in vitro, following collected first, and then injected into body for therapeutic effects after engineering, modification, or drug loading. In this way, exogenous biological vesicles served as delivery carriers, can not only deliver effective biological information of biological vesicles, but also convey therapeutic drugs. This exogenous biological vesicles‐based therapy has inherent advantages, including low immunogenicity, outstanding safety, in vivo targeting ability, and enhanced therapeutic effects.^[^ ^6^ ^]^ Therefore, it has been one of the most popular strategies of using exogenous biological vesicles in the treatment of various diseases.^[^ ^7^ ^]^

**Figure 1:**
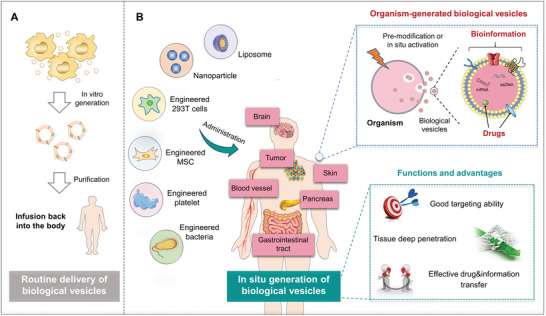
A) Routine delivery of exogenously generated biological vesicles and B) the novel strategy for in situ generation of biological vesicles via living organisms within body.

However, the strategy of using exogenous biological vesicles remains at the scientific and preclinical research stage at present, despite the fact that many efforts have been devoted to the development of biological vesicles. It cannot be denied that the traditional biological vesicles production process has become an important factor on restricting their clinical transformation (**Figure** **1**A). Although there are more and more methods to isolate and purify vesicles, none of them can fully meet clinical needs.^[^ ^8^ ^]^ Current isolation methods, such as size exclusion, ultra‐centrifugation, microfluidics, and immunoaffinity, cannot guarantee the yield and/or purity of biological vesicles.^[^ ^9^ ^]^ The isolation procedures usually involve many steps, which is quite laborious. The obtained number of biological vesicles is limited and the purity cannot be guaranteed, which limited their further applications. Furthermore, the isolated biological vesicles usually require further drug loading or functional modification. Extrusion, sonication, and organic solvents are often employed in the cargo‐loading process, during which the integrity and activity of biological vesicles may be destructed.^[^ ^10^ ^]^ As we all know, the activities of biological molecules, such as proteins and internal nucleic acids, and the integrity of biological vesicles both play a crucial role in their therapeutic effects. Therefore, using exogenous biological vesicles as therapeutic carriers is still facing great difficulties. A new kind of strategy to avoid ineffective work during in vitro separation and functionalization of biological vesicles needs to be laid out in the first place.

Many organisms, including cells^[^ ^11^ ^]^ and bacteria,^[^ ^12^ ^]^ could produce biological vesicles within body automatically, which is a normal physiological process. Under physiological conditions, biological vesicles, served as signal carriers, are involved in the homeostasis during the development of organism, such as cell differentiation. However, these automatically released biological vesicles only produced limited therapeutic effects in a pathological state, otherwise, the disease would not develop. Recently, it has been reported that exogenous stimulation can alter the generation and function of endogenous biological vesicles, endowing them with therapeutic properties.^[^ ^13^ ^]^ Therefore, in order to overcome the complex preparation process of exogenous biological vesicles, a new strategy has emerged, that is, using organisms to produce endogenous biological vesicles in situ as drug/bio‐information delivery carriers (Figure 1B). Unlike conventional and naturally occurred biological vesicles in vivo, this kind of organism‐produced endogenous vesicles is modified, stimulated, or functionalized in vivo by exogenous materials, and released in situ. Herein, autologous or transplanted organisms, such as cells or bacteria, were applied to release biological vesicles within the body. In order to endow these organism‐released biological vesicles with therapeutic functions, therapeutic nanoparticles or even cells were first injected into the body to functionalize organisms. Then, functional biological vesicles could be released into the body carrying both biological information and therapeutic drugs. For instance, intravenously administrated drug‐carrying fusogenic liposomes could fuse with tumor cells located at the outer layer of the tumor site, thereby releasing drug‐carrying biological vesicles.^[^ ^14^ ^]^ Deep penetration of the drug could then be achieved through the delivery of biological vesicles between cells. In addition, some agents that could promote the production of biological vesicles also were applied to act on cells and promote the formation and transport of biological vesicles in situ.^[^ ^15^ ^]^ Besides, transplanting functional cells or bacteria was another way of releasing drug‐carrying biological vesicles in the body. As illustrated in Figure 1B, these released biological vesicles have good targeting ability to the lesion sites, which has shown the potential to promote the deep penetration, and mediate the flow of biological information and drug delivery, so as to realize drug therapy and biological information delivery.

According to the type of endogenous biological vesicles, they can be divided into four categories: exosomes,^[^ ^16^ ^]^ platelet‐derived microparticles (PMPs),^[^ ^17^ ^]^ ApoBDs,^[^ ^18^ ^]^ and bacteria‐released OMVs.^[^ ^19^ ^]^ In this review, we will introduce the current development status and application prospects of organism‐produced biological vesicles, from the perspective of these four types of biological vesicles. We hope that this review could open up the application of biological vesicles in the field of biomedicine and shed light on current research.

## 2 Design Strategies of Using Organisms to Release Vesicles In Situ

**Figure 2:**
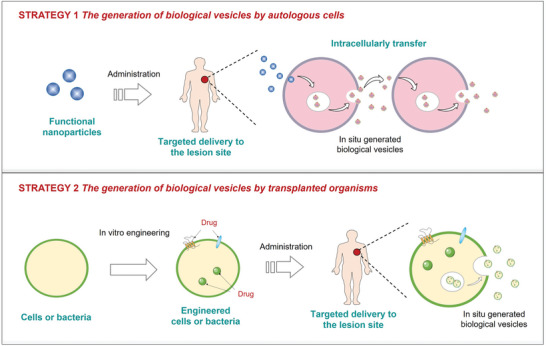
Design strategies of using organisms to release biological vesicles in situ.

As a novel strategy, there are not many reports about these organism‐produced endogenous biological vesicles. Biological vesicles released by mammalian cells are usually divided into three main subtypes, including exosomes, microvesicles, and ApoBDs.^[^ ^20^ ^]^ Exosomes are found to originate from endosomal compartment which is called the luminal vesicles of multivesicular bodies. Microvesicles are produced by outward budding from the cytoplasmic membrane, while ApoBDs arise during programmed cell death. Understanding the mechanism of organisms‐released biological vesicles and the functions of released biological vesicles in disease treatment could make us better design useful strategies for the medical application of biological vesicles. Based on the source and classification of organisms used to release biological vesicles, this in situ organism‐generated biological vesicles‐based strategy can be divided into two categories. One is the generation of functional vesicles by autologous cells in response to external stimuli in situ, such as nanoparticles carrying therapeutic drugs (**Figure** **2**). By targeting autologous cells in the body with nanomedicines, endogenous cells, such as tumor cells or T cells, could receive therapeutic drugs which either distributed on the cell membrane or dispersed in the cytoplasm. Under the stimulation of the drugs, these endogenous cells could release biological vesicles (exosomes or ApoBDs) from the other side. The released biological vesicles are more likely to carry drugs. Since biological vesicles could function as cell‐to‐cell communication, the released biological vesicles could be further internalized into other neighboring endogenous cells, either by membrane fusion or endocytosis. In turn, drugs are delivered to neighboring cells which could further release biological vesicles carrying drugs, enabling exchange of the drugs between endogenous cells and deep penetration of drugs in the tissue. The other way is to generate functional vesicles via transplanted organisms (Figure 2). In this way, genetically modified or functional organisms are transplanted into body and functional biological vesicles could be released under certain biological conditions. In the following part, we are going to explore the mechanisms and application directions of these two strategies.

### 2.1 The Generation of Functional Vesicles by Autologous Cells

As we have mentioned that almost all cells in the body could release biological vesicles, including exosomes and ApoBDs, under physiological conditions. Directly using autologous cells to endogenously release functionalized vesicles is a simple and effective method. Fusogenic liposomes carrying hydrophobic drugs on the membrane and hydrophilic content inside were often utilized to functionalize autologous cells in the body.^[^ ^14a^ ^]^ Then, autologous cells‐released vesicles carried drugs and performed therapeutic functions. Currently, this strategy was mainly applied in tumor treatment, especially in tumor deep penetration. It is well known that one of the biggest limitations of using nanomedicines to kill tumors is the difficulty of deep penetration at tumor sites. Due to the elevated pressure of tumor interstitial fluid, dense extracellular matrix, and closely packed tumor cells, the diffusion of molecules from the perivascular to the distal cells is hindered.^[^ ^21^ ^]^ Therefore, the extravasation of nanomedicine into tumor tissues is limited.^[^ ^22^ ^]^ For example, even oxygen molecules could only diffuse to a distance of 100–200 µm from the blood vessels.^[^ ^23^ ^]^ Most nanomedicine was located at the outer layer of the tumor after intravenous administration. On such occasion, the outer layer cells were first modified with therapeutic drugs and subsequently released drug‐contained exosomes or ApoBDs. Considering that exosomes are usually messengers of communication between cells,^[^ ^24^ ^]^ released exosomes or ApoBDs could be internalized into adjacent tumor cells which could further release biological vesicles carrying drugs. By doing so, the drug eventually passed from cell to cell via released biological vesicles, resulting in deep penetration.

### 2.2 The Generation of Functional Vesicles by Transplanted Organisms

Transplanting organisms into the body to actively release endogenous vesicles can avoid the loss caused by isolating vesicles in vitro. In this part, the organisms could be divided into two categories: cells and bacteria. With the development of cell therapy, cell transplantation techniques, including stem cells, platelets, and engineered cells, have been widely used in the treatment of various diseases. Usually, when the cells arrived at a certain environment, such as an inflammatory or tumor microenvironment, the corresponding vesicles could be triggered to release. For instance, genetically engineered mammalian cells were designed to consistently release exosomes for the delivery of cargo mRNA to the brain.^[^ ^15a^ ^]^ The released biological vesicles could arrive at recipient cells as autocrine, paracrine, or endocrine signaling. Specific binding of cellular receptors to vesicle‐related ligands facilitated communication between biological vesicles and cells.^[^ ^25^ ^]^ Biological vesicles have the ability to deliver or transfer their surface and internal contents into the membrane or cytoplasm of recipient cells, where these delivered molecules could display specific functions. It has been proved that these transplanted cells exerted a therapeutic effect in vivo by releasing microvesicles, exosomes, or ApoBDs.^[^ ^15^, ^26^ ^]^ Since the transplanted cells have anti‐inflammatory,^[^ ^27^ ^]^ drug‐loading, or special RNA functions,^[^ ^15a^ ^]^ the released vesicles were also endowed with corresponding functions. Another organism used for in situ releasing vesicles is bacteria that could release OMVs through outward budding. In formed OMVs, many functional biological molecules, including genetic materials, proteins, and antigens, were comprised. Therefore, OMVs have been widely used in immunotherapy.^[^ ^28^ ^]^ The release of OMVs in some parts of the body can be realized by certain modifications of bacteria. Of course, this strategy of remolding organisms in vitro, transplanting them into the body, and then releasing therapeutic vesicles in situ requires higher safety in the process. Relatively, it has higher controllability, effectiveness, and responsiveness and can better meet the needs of medical treatments.

## 3 Exosomes

Exosomes could be used to mediate information exchange between cells, by transferring cytoplasmic proteins, lipids, and genetic materials via membrane fusion. During the biosynthesis of exosomes, some specific molecules are incorporated and could be used as exosome markers, such as transmembrane tetraspanins CD63, CD9, and CD81.^[^ ^29^ ^]^ There has been considerable scientific interest in the potential use of exosomes as drug/information delivery vehicles. Various approaches to take advantage of the biochemical properties of exosomes as delivery vesicles were focusing on the isolation of exosomes and loading drugs in vitro, to achieve long circulation in the body, organ targeting, and therapeutic effect. However, both isolation of exosomes and engineering modification procedures might be laborious and result in exosomes damage.^[^ ^30^ ^]^ Recently, a new strategy of in situ exosome generation via organisms is increasingly being used for biological information transmission, drug delivery, and deep tumor delivery. According to the mechanism by which organisms produce exosomes, this section was divided into three directions: in situ generation of exosomes in tumor, endogenous enhancing/engineering exosomes via exogenous materials, and in situ generation of exosomes from engineered transplanted cells.

### 3.1 In Situ Generation of Exosomes in Tumor to Promote Drug Deep Penetration

**Figure 3:**
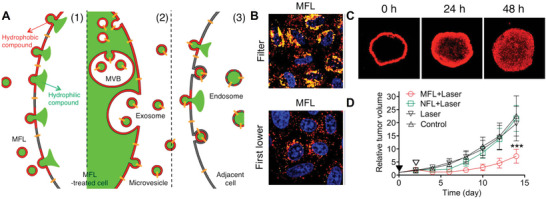
A) Schematic image showing that membrane vesicles including exosomes and microvesicles mediate delivery of hydrophilic and hydrophobic cargo to adjacent cells. B) Confocal microscopic images of HeLa cells in the transwell filter and the first lower chamber. C) Confocal microscopic images of HeLa tumor spheroids treated with ZnPc‐loaded liposomes. D) Tumor growth inhibition by photodynamic therapy with exosomes‐mediated penetration of ZnPc in CT26 tumors. A–D) Reproduced with permission.^[^ ^14a^ ^]^ Copyright 2015, The American Chemical Society.

Although exosomes have been widely used in the treatment of tumors, the distribution of exosomes within tumors is still in urgent need of improvement. To address the problem, in 2015, J. Park^[^ ^14a^ ^]^ first reported that by using exogenous compounds‐loading synthetic membrane fusogenic liposomes (MFLs), it was possible to package external compounds into exosomes efficiently. The original parental cells could first fuse with MFLs, generating engineered cells with modified surface membrane and cytoplasmic content. Then, new exosomes released from engineered cells were actively transferred to neighboring cells. Since exosomes derived from engineered cells contained portions of cytosol and membrane of original cells, the exogenous compounds carried by MFLs could be transferred to the newly generated exosomes. Using hydrophobic DiI and hydrophilic calcein as model drugs, they found that MFLs could successfully deliver DiI and calcein to the surface membrane or cytoplasm of cancer cells via membrane fusion, respectively (**Figure** **3**A). Moreover, DiI and calcein could be translocated to exosomes from cells treated with MFLs and further transferred to neighboring cells via exosomes mediated communication in transwell systems (Figure 3B). However, due to the degradation in endosomes/lysosomes, the number of hydrophilic compounds transferred to adjacent cells attenuated with each transfer, while the transfer efficiency of hydrophobic compounds remained stable, suggesting that this exosome‐mediated transportation was more suitable for hydrophobic compounds. In a multicellular tumor spheroid model, hydrophobic photosensitizer zinc phthalocyanine carried by MFLs could penetrate deeply into the tumor spheroid via successive multiple cell uptake and subsequent new exosomes generation, thereby achieving efficient in vivo tumor inhibition under the photodynamic therapy (Figure 3C,D). For the application of this system in vivo, J. Park has demonstrated that MFLs could promote deep penetration of the drug at the tumor site in fluorescence distribution experiments and histological analysis. But how the initial MFLs targeted the tumor has not been discussed. Of course, the enhanced permeability and retention (EPR) effect may increase the accumulation of MFLs at the tumor site, but the exact reason is not illustrated. This work provided a proof of concept that in situ engineering generation and mediating the tumor intercellular penetration using exosome could be an intercellular translocation system.

In addition to cell modification using MFLs, W. Xue^[^ ^31^ ^]^ reported another way of modifying tumor cells through worm‐like nanocell mimics (WP@R‐EM) which was composed of tumor‐targeted erythrocyte membrane (EM) as cloak and a worm‐like BSA‐siRNA nanoparticle (WP) as the core. Moreover, lipidated cyclic arginine–glycine–aspartate (cRGD) was inserted into EM to endow the system with targeting function. By incubation with remodel *α*v*β*3 integrin‐overexpressed cancer cells, WP@R‐EM could fuse with cell membrane and the WP entered directly into the cell interior. By analyzing the membrane fusion mechanism between WP@R‐EM and cancer cells, it was found that cRGD‐mediated targeting played an important role in cellular uptake and membrane fusion, while the dyes labeled EM and WP were highly overlapped in the worm‐like nanocell mimics without cRGD modification. As exosomes could deliver lipids and biological cargoes from parental cells to adjacent cells, it was confirmed that tumor cells engineered with DiO containing WP@R‐EM could effectively transfer DiO to neighboring cells via exosomes secretion. Contrary to what J. Park reported,^[^ ^14a^ ^]^ however, the secreted exosomes were also efficient at delivering cytoplasmic materials engineered by WP@R‐EM, as siRNA released from WP@R‐EM could be packaged into exosomes and successively delivered to neighboring or distant cells. The reason for this difference was not explained in this article. We speculated that it might be attributed to the difference between micro/small molecules. Similar to the excellent in vitro performance of WP@R‐EM in engineering tumor cells, WP@R‐EM carrying two therapeutic cargoes, DIR for photothermal therapy and VEGF‐downregulating siRNA, showed exceptional inhibition on both primary tumor and tumor metastasis, as the spontaneously generated exosomes in tumor tissue could promote deep penetration of two therapeutic drugs. Contrary to traditional methods that were used to isolate exosomes as drug carriers, exosomes in situ generated in the tumor microenvironment can be utilized and reconfigured directly, carrying exogenous therapeutics across multiple cell layers through inherent intercellular migration mechanisms.

### 3.2 In Situ Generation of Exosomes in Tumor to Facilitate Cooperative Targeting Strategy

In situ generation of exosomes within tumor to improve the drug delivery efficiency has also been reflected in collaborative targeting therapy. The so‐called cooperative targeting strategies referred to the first exogenous addition of surface receptors/reactive groups on the surface of tumor cells.^[^ ^32^ ^]^ Subsequently, therapeutic/imaging drugs with corresponding matched binding groups were administrated to target labeled tumor cells. However, similar to conventional drug delivery, pre‐delivery and pre‐modification of targeted groups at tumor sites were also limited by inadequate efficiency. Therefore, enhancing the distribution of targeted receptors in tumors by using cell‐in situ‐produced exosomes as delivery agents has been utilized as a useful strategy.

**Figure 4:**
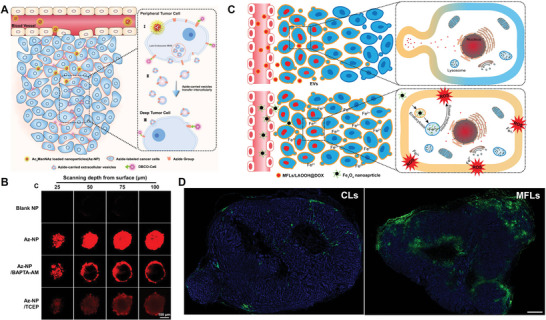
A) Schematic diagram of nanoparticle‐mediated metabolic labeling of azide groups on the perivascular tumor cell membrane and intercellular transfer of the azide groups to deep tumor cells through extracellular vesicles for enhanced tumor targeting and penetration of DBCO‐Ce6 by a bioorthogonal click reaction. B) CLSM observation of 4T1 multicellular spheroids incubated with various groups. C) Schematic design of MFLs‐based anticancer drug delivery for treatment of malignant tumors. D) Fluorescence images of 4T1 tumor sections after intravenous injection of CLs or MFLs. A,B) Reproduced with permission.^[^ ^34^ ^]^ Copyright 2020, Elsevier. C,D) Reproduced with permission.^[^ ^14c^ ^]^ Copyright 2019, The American Chemical Society.

In J. Park's work,^[^ ^14b^ ^]^ the ability of exosomes to transmit hydrophobic lipids among cells was utilized to re‐distribute synthetic receptor‐lipids (SR‐lipids) within tumor. It was reported that nanomedicine may arrive at tumor sites through EPR effect, but most of the time it mainly distributed in the outer layer of the tumor.^[^ ^33^ ^]^ Therefore, MFLs carrying SR‐lipids could fuse with plasma membranes of cells located in the outer layer of the tumor, where SR‐lipids containing exosomes were released and autonomously penetrated layer‐by‐layer into the tumor via intercellular transport. It ultimately led to the distribution of SR‐lipids throughout the whole tumor, which facilitated the distribution of therapeutic agents on tumor cell surface via targeting SRs. What's more important, when SR‐lipids containing MFLs accumulated in the mononuclear phagocytic system, such as liver, most of them underwent lysosomal degradation pathway, other than releasing exosomes. Therefore, there was minimal accumulation of SRs in liver. In this way, the target drugs could be selectively delivered to tumor cell membrane. Using biotin–streptavidin as receptor‐binding pair, the distribution of biotin‐lipids and streptavidin‐photosensitizer at tumor was significantly improved, so as to achieve excellent photodynamic therapy effect. In addition to phospholipid‐mediated modification of cell membranes, metabolic glycoengineering was also a popular way to realize cancer cell membrane surface engineering. Combining with in vivo bioorthogonal click reaction, this cooperative targeting strategy has exhibited great advantages in improving the efficiency of tumor targeting. In J. Wang's work,^[^ ^34^ ^]^ taking advantage of exosomes released by perivascular tumor cells, it realized the deep penetration of azide‐containing ligands within tumor (**Figure** **4**A). In simple terms, metabolic precursors (Az‐NPs) were first administrated to label perivascular tumor cells surface with azide groups via metabolic engineering. Azide ligands were then autonomously transported deeply into tumor, accompanied by in situ generation and intercellular transportation of exosomes. In the end, improved tumor penetration of dibenzocyclooctyne‐modified chlorin e6 was achieved through bioorthogonal click reaction and the effect of photodynamic therapy on tumor has been improved accordingly (Figure 4B).

Similarly, in Z. Zhang's work,^[^ ^14c^ ^]^ exosomes in situ released by tumor cells have also been used to improve the drug deep tumor penetration (Figure 4C). Linoleic acid hydroperoxide (LAOOH), a ROS‐generated agent, was incorporated into MFLs (MFLs/LAOOH@DOX) which also contained chemotherapy drug DOX inside. Similar with what was observed by J. Park,^[^ ^14a^ ^]^ with the assistance of exosomes, hydrophobic LAOOH could migrate gradually to adjacent cells and ultimately spread throughout the whole tumor, while DOX, as hydrophilic drug distributed within cytoplasm, only demonstrated limited tumor penetration (Figure 4D). Therefore, administrating DOX alone was not enough to exert efficient anti‐tumor effect. To improve the therapeutic effect, Fe_3_O_4_ nanoparticles were subsequently administrated and released Fe^2+^ in the tumor microenvironment under acidic conditions. As Fe^2+^ could reach the interior of tumor easily to catalyze LAOOH located on the cell membrane, abundant ROS was generated through the Russell mechanism, which in end resulted in tumor apoptosis. This exosomes‐mediated lipid exchange strategy facilitated the sequential intercellular delivery system with enhanced ROS‐dependent antitumor efficacy and tumor distribution simultaneously. In general, in this exosome‐mediated cooperative targeting strategy, targeting groups first penetrated deeply and spread all through the whole tumor via exosomes in situ generation and cell‐to‐cell transportation. The therapeutic drugs labeled with corresponding match groups could be easily navigated deep into the tumor and exert therapeutic effect. This strategy is currently mostly used for deep penetration of tumors.

### 3.3 Endogenous Enhancing/Engineering Exosomes via Exogenous Materials

In theory, the outer layer of tumor cells was first exposed to the drugs/nanosystems and secreted endogenous exosomes carrying corresponding drugs autonomously. These generated exosomes actively migrated to the neighboring layers of cells, gradually penetrated layer‐by‐layer into the interior of the tumor, and then killed them. However, in most cases, the inadequate exosomes secretion efficiency within tumor or limited therapeutic effect of exosome confined the effectiveness of this strategy in tumor therapy. Therefore, the use of exogenous means to enhance the secretion or engineering modification of exosomes by tumor cells could be a valid strategy to improve the delivery of drugs in the tumor.

**Figure 5:**
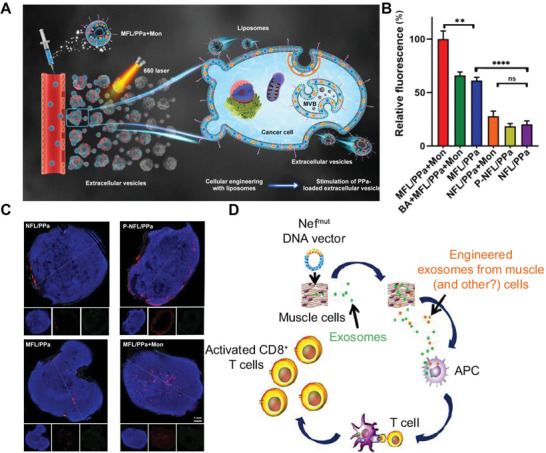
A) Schematic representation of MFL/PPa+Mon and the amplified penetration of PPa by Mon‐mediated EV release stimulation. B) Fluorescence quantification of PPa in EVs derived from different groups. C) Fluorescence images of 4T1 tumor sections taken after 48 h of intravenous injection with different groups. D) Mechanism of CTL activation induced by inoculation of Nef^mut^‐based DNA vectors. A–C) Reproduced with permission.^[^ ^13a^ ^]^ Copyright 2022, The American Chemical Society. D) Reproduced with permission.^[^ ^26^ ^]^ Copyright 2017, Dove Press.

In W. Liang's work,^[^ ^13a^ ^]^ monensin, a polyether Na^+^ ionophore for stimulating exosomes‐releasing,^[^ ^35^ ^]^ was applied as an exosomes secretion stimulant to improve the exosome‐mediated tumor penetration of nanomedicines (**Figure** **5**A). Generally, adopting a similar strategy to J. Park,^[^ ^14a^ ^]^ monensin and photosensitizer PPa were co‐loaded in MFLs (MFL/PPa+Mon). It was confirmed that adding PPa and monensin into MFLs would not influence their membrane fusion ability. As the reagent for photodynamic therapy, the wide distribution of PPa at tumor site directly determined its tumor‐killing effect. With the intervention of monensin, both the amount of exosomes production and the quantity of PPa incorporated in exosomes were significantly promoted (Figure 5B). Both in the in vitro 4T1 tumor spheroid model and in vivo solid tumor, PPa exhibited the best migration level in the tumor treated with MFL/PPa+Mon. Moreover, exosomes isolated from MFL/PPa+Mon‐treated cells were injected into tumor‐bearing mice and proved to have the best tumor penetration, which verified the exosome‐mediated deep penetration (Figure 5C). Collectively, the results verified that using monensin to stimulate in situ release of exosomes played an important role in enhancing tumor penetration. In fact, studies have shown that there were a variety of ways to promote exosome production by cells in situ, including stress or injury to cells, regulating small GTPases, heat shock protein 90, cortactin, and Ca^2+^ (like monensin).^[^ ^36^ ^]^ Therefore, in order to make better use of exosome‐mediated deep tumor penetration, the combination of promoting in situ generation of exosomes and drug delivery may be the potential way for future research.

At present, exosomes with immunogenicity are potential cancer vaccines, owing to the incorporation of antigens. The immunogenicity of exosomes mainly was derived from their parenteral cells and sometimes, insufficient immunogenicity of exosomes was not enough to stimulate an effective immune response, owing to the limited amounts of antigens. Except for commonly used in vitro modification method to endow exosomes with foreign antigens,^[^ ^37^ ^]^ in vivo engineering autologous cells to generate exosomes expressing specific antigens has gained great interest. In M. Federico's work,^[^ ^26^ ^]^ they found that intramuscular inoculation of mice with DNA vectors which expressed protein antigens Nef^mut^/E7 could enable muscle cells constitutively release exosomes (Figure 5D). In these released exosomes, large amounts of antigens were incorporated and therefore, a specific cytotoxic CD8^+^ T lymphocytes response was elicited against both Nef and E7. This method provided a way of in situ generation and engineering functional exosomes as anti‐tumor vaccines.

### 3.4 In Situ Generation of Exosomes from Engineered Transplanted Cells

Except for using autologous cells to generate exosomes in situ, engineered transplanted cells also could be applied to release therapeutic exosomes in body. M. Fussenegger et al^[^ ^15a^ ^]^ reported that engineered mammalian cells implanted in patients were capable of secreting therapeutic exosomes to treat Parkinson's disease effectively. In brief, a set of devices called exosomal transfer into cells was transfected into HEK‐293T cells. This transfection endowed cells with enhanced exosomes production and specific mRNA package within exosomes. As long as the engineered producer cells were implanted into living body, therapeutic cargo mRNA could be packed into exosomes and delivered to brain, exerting the effect of reducing neurotoxicity and neuroinflammation. Currently, transplanted cells have been widely used as drug/nanoparticles delivery carriers to release therapeutics at targeting site in situ.^[^ ^38^ ^]^ However, such kind of using transplanted cells as an exosomes production source was barely reported. In fact, this method could be expanded into in situ releasing of exosome‐coated nanoparticles/drugs. The ability of using the released nanoparticles as a drug reservoir and exosomes’ biological information might be a better therapeutic strategy.

## 4 Platelet‐Derived Microparticles

Platelets are an essential part of blood with disc‐shape and the size ranging from 1 to 3 µm.^[^ ^39^ ^]^ Their circulation time in the body is 8 to 10 days. At rest, platelets circulate in the blood vessels like sentinels, while in the event of vascular damage (such as stroke, myocardial infarction, and surgery), platelets are activated rapidly and accumulate at the site of damage. Notably, the activation would induce the release of small vesicles or granules from platelets to communicate with other cells and exert their biological functions.^[^ ^40^ ^]^ Inspired by the unique vesicle release behavior, a variety of drug‐modified platelets were constructed for disease treatments.^[^ ^39b^ ^]^ The drugs can be inserted, conjugated, swallowed, or genetically engineered into platelets, and released from platelets in the form of PMPs upon activation. As a drug delivery platform, the engineered platelets have the advantages of fast metabolism, low side effects, disease targeting ability, and triggered drug release behavior.^[^ ^41^ ^]^ Such inherent propensity and structure alteration ability would regulate the biodistribution of loaded drugs and meanwhile exert synergic effect through the release of PMPs or granules. In addition, in order to reinforce the disease‐targeting ability of drug‐loaded platelets and corresponding vesicles, many intelligent strategies have been proposed for precise disease treatment.

### 4.1 In Situ Generation of Vesicles by Drug‐Loading or Conjugated Platelets

**Figure 6:**
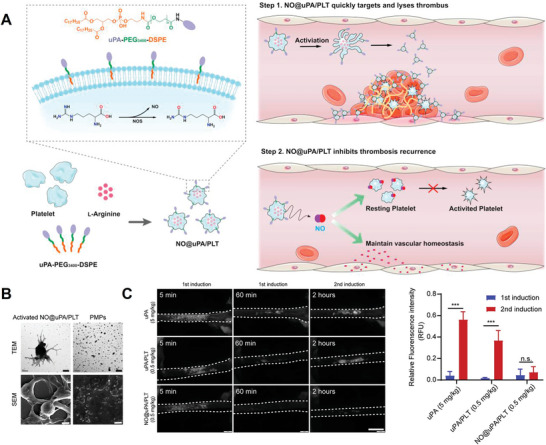
A) Schematic action mechanism of NO@uPA/PLTs for targeting thrombus and inhibiting thrombosis recurrence. B) The TEM and SEM images of activated NO@uPA/PLTs and the release vesicles (PMPs). Scale bar = 1 µm. C) The treatment efficiency of uPA (5 mg kg^−1^), uPA/PLTs (0.5 mg kg^−1^), and NO@uPA/PLTs (0.5 mg kg^−1^) and the quantitative results of rhodamine 6G fluorescence intensity in different groups (*n* = 3). *p < 0.001. n.s., not significant. A–C) Reproduced with permission.^[^ ^43^ ^]^ Copyright 2022, Elsevier.

Platelets are the basis of thrombotic diseases. When vascular lesions occur, platelets will adhere to endothelial cells, accumulate, activate, and form thrombosis in the blood vessels, resulting in vascular embolism and a series of thrombotic diseases.^[^ ^42^ ^]^ Given the central role of platelets in thrombosis, M. Liu et al.^[^ ^43^ ^]^ integrated a platelet drug delivery platform to target thrombus for treating pulmonary embolism and FeCl_3_‐induced carotid artery thrombosis in mice, in which platelets acted as the delivery vehicle, urokinase (uPA) as the targeting group, and nitric oxide (NO)‐generated arginine (Arg) as the therapeutic drug to inhibit re‐embolism (**Figure** **6**A). Once the platelet‐based platform (NO@uPA/PLT) reached the thrombus site and was activated by thrombin, they would transform into an amorphous shape, which was exhibited in the TEM and SEM results, and generate PMPs with a size of about 200 nm (Figure 6B). In the mice with thrombin‐induced pulmonary embolism, NO@uPA/PLT demonstrated significant lung targeting ability, evidenced by the 5.4‐fold higher lungs fluorescence intensity in model mice compared with that in non‐model mice. In the treatment results of mice with carotid arterial thrombosis, high dosage of free uPA (5 mg kg^−1^) and low dosage of NO@uPA/PLTs (0.5 mg kg^−1^) both demonstrated good in vivo therapeutic effect upon first FeCl_3_ induction, while free uPA (5 mg kg^−1^) failed to prevent the re‐embolism upon secondary FeCl_3_ induction (Figure 6C). Surprisingly, NO@uPA/PLTs (0.5 mg kg^−1^) could prevent the re‐embolism in blood, exhibiting the effectiveness of this strategy.

In addition to the aforementioned wound‐targeting ability, platelets could also interact with and be activated by tumor cells or circulating tumor cells.^[^ ^44^ ^]^ It is reported that platelets can induce dendritic cells (DCs) maturation and boost the immune response of T cells.^[^ ^45^ ^]^ In 2017, Z. Gu's team^[^ ^46^ ^]^ was pioneered to conjugate PDL1, a kind of immune checkpoint inhibitor antibody, on the surface of platelets for preventing the recurrence and metastasis of tumor after surgery. The PD‐L1‐conjugated platelets (P‐aPDL1) could be directed to and enriched in the site of injury by their inherent propensity to wound, thereby avoiding the systemic toxicity of free PDL1. The fluorescence in P‐aPDL1 treated mice was 9.4‐fold higher than that in free aPDL1 treated mice. Interestingly, they found that a lot of PDL1‐conjugated derivative PMPs was produced upon activation at the surgical site or interaction with residual tumor cells or circulating tumor cells in blood. aPDL1 was relatively stable in non‐activated platelets and could be largely released after activation. The activated platelets were branching in electron microscopy results. In addition, the accumulated platelets could attract immune cells and boost immune killing of tumor cells at the surgical site. This strategy demonstrated significantly delayed tumor recurrence and prolonged survival in B16F10 tumor‐bearing mice. Subsequently, they further combined this strategy with thermal ablation to achieve remarkably diminished or even cleared tumor recurrence and metastasis.^[^ ^47^ ^]^ Except for the insertion or conjugation method for the loading of free drug, drug‐loaded nanoparticles could also be integrated into platelets, including nanocomplex,^[^ ^48^ ^]^ nanogels, nanodiamond,^[^ ^49^ ^]^ and liposome.^[^ ^50^ ^]^

### 4.2 In Situ Generation of Vesicles by Genetically Engineered Platelets

**Figure 7:**
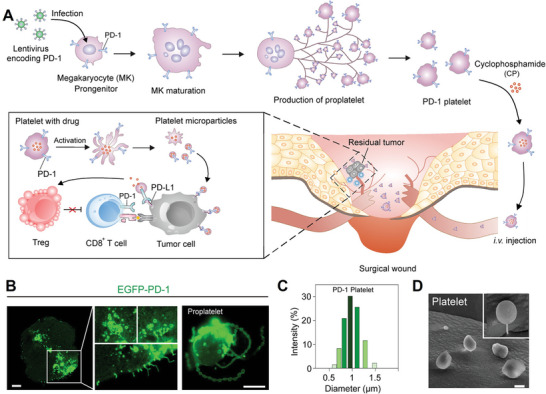
A) Schematic diagram of PD‐1 expressing process and action mechanism of PD‐1‐engineered platelets. B) The morphology of PD‐1 proplatelets from L8057 cells. Scale bar = 10 µm. C) Size distribution and D) morphology of PD‐1‐expressing platelets. Scale bar = 1 µm. A–D) Reproduced with permission.^[^ ^52^ ^]^ Copyright 2018, The American Chemical Society.

In fact, platelets are small cytoplasmic fragments of mature megakaryocytes (MK) in bone marrow hematopoietic tissue.^[^ ^51^ ^]^ Therefore, they are non‐nucleated and terminally differentiated cells, and it is difficult to control their biological information by means of genetic engineering. In view of the production source of platelet, Z. Gu et al.^[^ ^52^ ^]^ prepared a kind of programmed cell death protein 1 (PD‐1) expressing platelet through genetic engineering of MK progenitor cells L8057 (**Figure** **7**A). After being infected by lentivirus and screened by puromycin, stable EGFP‐PD‐1 expressing L8057 cells were established, which would bud and extend PD‐1 proplatelets after 6 days stimulation by 500 nm of phorbol 12‐myristate 13‐acetate (Figure 7B). The extended proplatelets could be disbanded from MK progenitor cells, generating PD‐1‐expressing platelets (Figure 7C,D). Similar to the aforementioned platelets, these PD‐1 expressing platelets could also release the small size of PMPs and demonstrate good therapeutic effects in inhibiting tumor recurrence post‐surgery. Moreover, they could also serve as a drug carrier for cyclophosphamide and this combination resulted in prolonged survival of tumor‐bearing mice.

Platelets can be applied in other immune‐associated diseases.^[^ ^53^ ^]^ In 2020, Z. Gu et al.^[^ ^53a^ ^]^ further developed PD‐L1‐engineered platelets to modulate the PD‐1/PD‐L1 signal axis for protecting pancreatic *β* cell in new‐onset type 1 diabetes mice. The destruction of insulin‐secreting *β*‐cells by islet specific autoreactive T cells is major pathogenesis of type 1 diabetes.^[^ ^54^ ^]^ In a mouse model of type 1 diabetes, PD‐L1‐engineered platelets could actively target inflammatory pancreatic tissues, which would interact with T cells and inhibit the activity of autoreactive T cells in the pancreas, thereby maintaining the integrality of insulin‐secreting *β*‐cells. Around 75% of mice recovered from the new‐onset type 1 diabetes after PD‐L1‐expressing platelets’ treatment. Mechanism studies demonstrated that the therapeutic effect of PD‐L1 expressing platelets was linked to the decreased percentage of CD8^+^ T cells and cytotoxicity T Cells (GzmB^+^CD8^+^ and IFN‐*γ* ^+^CD8^+^), and increased proportion of regulatory T cells (Tregs) for maintaining immune tolerance microenvironment.

### 4.3 Methods to Alter the Biodistribution of Platelets and Platelets‐Released Vesicles

Precise targeting of lesion locations is the primary mission of drug delivery system. Scientists have developed many ways to alter drug distribution for the platelets cell delivery systems and in situ produced vesicles. Current strategies were mainly focused on altering the distribution of parental platelets, including the use of vascular injury substances to enhance wound targeting ability,^[^ ^55^ ^]^ the coupling of bone marrow targeting cells to change the in vivo fate,^[^ ^56^ ^]^ and the use of gel systems for achieving sustained drug release.^[^ ^57^ ^]^

**Figure 8:**
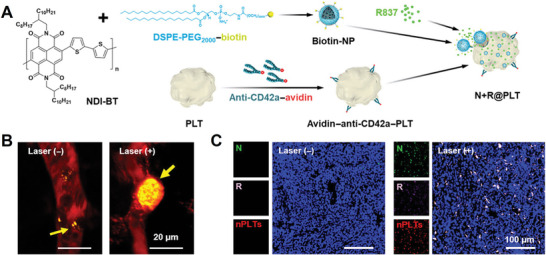
A) The preparation process of photothermal nanoparticles and immune agonists co‐loaded platelets. B) In vivo multiphoton animal images of tumor vessels in mice treated with drug‐loaded platelets before or after irradiation. Red, vessels; yellow, drug‐loaded platelets. C) Tumor fluorescence images of nanosized platelets vesicles with or without laser irradiation. A–C) Reproduced with permission.^[^ ^55b^ ^]^ Copyright 2022, American Association for the Advancement of Science.

One conventional strategy for changing drug distribution is to enhance the targeting efficiency of delivery systems. On account of the targeting mechanism of platelets, Z. Gu's group^[^ ^55a^ ^]^ developed a novel method to enhance platelet targeting in tumors by increasing the degree of vascular injury. By combining with vascular injury agents, Vadimezan, the targeting efficiency of PDL1‐conjugated platelets at 4T1 lung metastatic tumor sites can be increased by three and ten times, respectively, compared with single PDL1‐conjugated platelets and free aPD‐L1. Moreover, 50% of mice survived at 60 days after tumor incubation in the combined group. Similarly, in the same year 2021, Y. Lv et al.^[^ ^55b^ ^]^ found that phototherapy could improve the targeting efficiency of drug‐loaded platelets, achieving combinational photothermal‐immunotherapy (**Figure** **8**A). In this system, photothermal nanoparticles and immune agonists, serving as the bullets, were loaded into platelets. Under the irradiation with near‐infrared light (0.65 W cm^−2^), acute vascular injury at tumor site could induce the aggregation and activation of drug‐loaded platelets, resulting in a positive feedback loop and the formation of thrombus at the tumor site, serving as the “ammunition” of photothermal nanoparticles and immune agonists (Figure 8B). At the same time, the activated platelets can release nanosized platelet vesicles for facilitating the extravasation into tumor vessel and penetration of loaded drugs deep into the tumor tissue (Figure 8C). The synergy effect of photothermal‐immunotherapy was fully verified in nine murine models and a sophisticated patient‐derived xenograft tumor model.

The application of homologous targeting ability of tumor cells can also alter the distribution of drug‐loaded platelets. Z. Gu's team^[^ ^56^ ^]^ proposed “combined cell delivery” technology to treat leukemia, in which hematopoietic stem cells (HSC) and PD‐1 conjugated platelets were joined together by means of a chemical connector. In this system, HSC acted as a navigation system for bone marrow homing, the platelets as a van to deliver drugs, and the PD‐1 as the cargo to treat disease. When injected intravenously, HSC could direct platelets to the bone marrow. The fluorescence level in PD‐1 or PD‐1‐platetelts conjugated HSC was 25‐fold higher than that in other groups without the connection of HSC. The platelets in bone were then activated to release PD‐1 antibodies in the form of vesicles that can be used as immunotherapy to activate the body's immune T cells and kill leukemia cells. About 87.5% of mice treated with the PD‐1 decorated combined cells have survived for over 80 days. Subsequently, Gu's team^[^ ^57^ ^]^ fabricated a biodegradable hydrogel reservoir for in vivo storage of CAR T cells and PD‐L1 conjugated platelets, continuously releasing immune checkpoint inhibitor and CAR T cells at the site of tumor resection. This system can improve the efficacy of CAR T cells in solid tumors, effectively eliminate residual tumor cells, and prevent tumor recurrence.

## 5 Apoptotic Bodies

Apoptosis is a kind of physiologically programmed cell death that occurs in many different cells. It plays an important role to regulate cell populations in developmental and adult tissues. Vesicular ApoBDs could be released during cell apoptosis, with particle sizes ranging from 500 to 5000 nm and variable structures and compositions. Unlike exosomes (30–100 nm) and microvesicles (100–1000 nm) which are secreted along with normal cellular activity, ApoBDs are generated only with programmed cell death. Except for some general makers (calreticulin, ALIX, and TSG101) which are expressed on ApoBDs, apoptotic products, such as cleaved caspase 3 and enriched phosphatidylserine are also inherited in ApoBDs.^[^ ^58^ ^]^ In addition, to distinguish ApoBDs from cell debris of the same size (1–5 µm), microscopies including confocal laser scanning microscope, transmission electron microscopy, and scanning electron microscopy, could be applied to detect the presence of ApoBDs with intact spherical structures. ApoBDs typically contain genetic materials, histones, and intact or fragmented organelles, owing to their large size. Dying cells could communicate with neighboring cells via the soluble and signal mediators encapsulated in ApoBDs. During normal physiological activities in vivo, most ApoBDs are phagocytosed and cleared by macrophages.^[^ ^3^, ^59^ ^]^ Besides, the rapid proliferation of tumor cells usually results in nutrient deprivation in the tumor microenvironment. Tumor cells could obtain supplementary nutrients through macropinocytosis of macromolecules from the microenvironment, such as ApoBDs. Therefore, ApoBDs released by tumor cells can be internalized into neighboring tumor cells and contribute to delivering drugs or biological messages.^[^ ^24^ ^]^ Therefore, triggering the release of ApoBDs could be used as a potential strategy to achieve in vivo transmission of biological information and drugs, especially in the tumor microenvironment. The in situ triggering release of ApoBDs can be divided into two categories. One is by transplanting cells into the body, mostly mesenchymal stem cells (MSCs). Transplanted cells could regulate physiological functions by releasing ApoBDs in vivo. The other is to deliver drugs into the body, triggering the release of ApoBDs by existing cells in the body, and then utilizing the bioinformation and drug delivery function of ApoBDs.

### 5.1 Apoptotic Bodies Produced by Transplanted Cells

**Figure 9:**
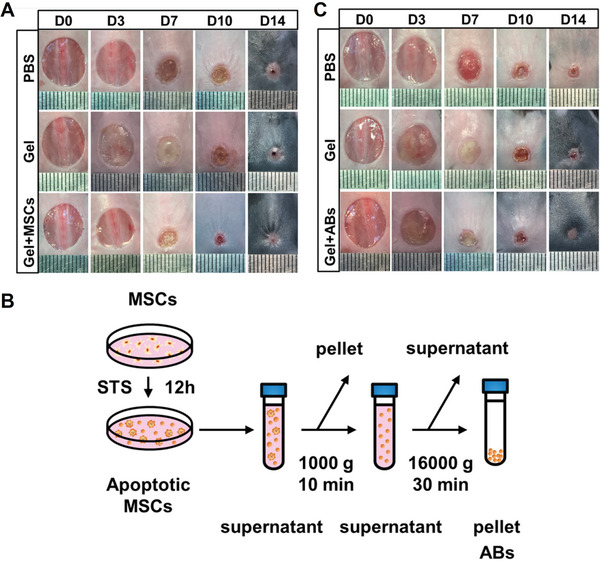
A) Representative photographs of cutaneous wounds in PBS, gel, and gel+MSCs groups at different time points during the wound healing procedure. B) The schematic graph shows the protocol of isolation of ApoBDs from MSCs. C) Representative photographs of cutaneous wounds in PBS, gel, and gel+ApoBDs groups at different time points during the wound healing procedure. A–C) Reproduced with permission.^[^ ^70^ ^]^ Copyright 2020, Springer Nature.

MSCs transplantation has been widely used in the treatment of a variety of diseases, such as autoimmune diseases,^[^ ^60^ ^]^ myocardial infarction,^[^ ^61^ ^]^ diabetes,^[^ ^62^ ^]^ kidney injury,^[^ ^63^ ^]^ and cirrhosis.^[^ ^64^ ^]^ Currently, the general consensus on using MSCs as one the useful therapeutic strategies was based on their ability to secrete massive chemokines, cytokines, and even subcellular particles.^[^ ^65^ ^]^ However, the exact in vivo metabolic fate of these transplanted MSCs after infusion remained to be unknown. Interestingly, it was found that in many pieces of research, transplanted MSCs underwent apoptosis not before long after being injected into the body.^[^ ^66^ ^]^ And what's more important, the self‐initiated apoptosis of MSCs played an important role in the regulation of inflammation. For example, Yan Jin et al.^[^ ^67^ ^]^ administrated human mesenchymal stem cells (hMSCs) into a rabbit model to heal the hypertrophic scar. By analyzing the underlying mechanisms, they found that the survival rate of hMSCs after injection was significantly reduced and resulted in extensive apoptosis. Meanwhile, apoptotic hMSCs were proved to secrete a large number of anti‐inflammatory proteins, via the activation of caspase‐3. Not coincidentally, in a murine model of graft‐versus‐host disease, F. Dazzi et al.^[^ ^68^ ^]^ also demonstrated that infused MSCs exerted immunosuppressive effects through perforation‐dependent apoptosis which was triggered by recipient cytotoxic cells. R. Henscher et al.^[^ ^27^ ^]^ reported that apoptotic and phagocytotic markers were quickly found on the surface of infused MSCs and these apoptotic MSCs would further accumulate in the lung and liver. T. S. P. Heng et al.^[^ ^66^ ^]^ confirmed that the response of the host to apoptotic MSCs is the key to the therapeutic effects of MSCs. Alternatively, another theory was that MSCs‐induced immunoregulation involved the apoptosis of T cells.^[^ ^69^ ^]^ Although in vivo apoptosis of MSCs has been mentioned in the above articles, the role of ApoBDs in MSCs has not been identified. Only a few studies have definitively pointed out that many ApoBDs generated during MSCs apoptosis could result in immune tolerance to ameliorate autoimmune disorders. In Y. Jin's research,^[^ ^70^ ^]^ the therapeutic effect of infused MSCs on wound healing in mice was tested and a mass of apoptosis of MSCs was observed (**Figure** **9**A). To verify the therapeutic effect of apoptotic MSCs, they extracted ApoBDs and demonstrated that ApoBDs could speed up the healing process of skin wounds (Figure 9B,C). Besides, in a myocardial infarction model, they also found that infused MSCs could undergo extensive apoptosis and release ApoBDs to promote angiogenesis and improve cardiac recovery via regulating autophagy in the recipient endothelial cells.^[^ ^15b^ ^]^

### 5.2 Apoptotic Bodies Triggered by Exogenous Nanoparticles

**Figure 10:**
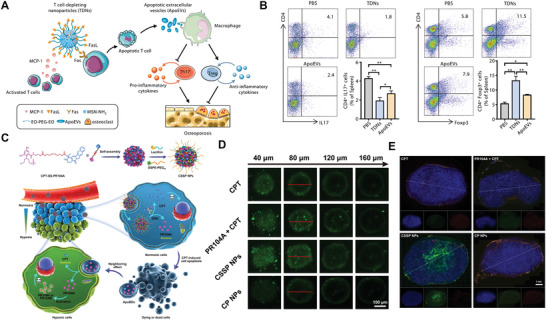
A) Schematic illustration of T cell‐depleting nanoparticles and their therapeutic effects on osteoporosis through induction of T cell apoptosis and regulation of the Tregs/Th17 balance. B) Frequencies of CD4^+^/IL17^+^ Th17 cells and CD4^+^/Foxp3^+^ Tregs at 8 weeks post‐ovariectomy. C) Fabrication of self‐assembled CSSP NPs and the illustration of CSSP NPs enhancing drug penetration and whole tumor destruction through ApoBD‐mediated neighboring effect. D) Z‐stack images for drug penetration in 3D tumor spheroids. E) Fluorescence images of 4T1 tumor sections of CPT penetration after intravenous injection of various formulations. A,B) Reproduced with permission.^[^ ^71^ ^]^ Copyright 2021, Elsevier. C–E) Reproduced with permission.^[^ ^72^ ^]^ Copyright 2021, American Association for the Advancement of Science.

In addition, directly triggering in vivo apoptosis of existing cells, such as T cells or tumor cells, and in situ releasing ApoBDs, could also be used to regulate physiological functions or deliver drugs.^[^ ^13b^ ^]^ ApoBDs played an important role in regulating immune tolerance. It has been reported that in estrogen deficiency‐associated bone loss, T cells were usually overactivated, and secreted many pro‐osteoclastogenic factors to accelerate bone resorption, resulting in bone loss. Therefore, in Y. Jin's group,^[^ ^71^ ^]^ they proposed a nanoparticle‐inducing T cell depletion method to re‐establish immune homeostasis in estrogen deficiency induced‐bone homeostasis. The proposed nanoparticles were composed of mesoporous silica nanoparticles loaded with monocyte chemotactic protein‐1 (MCP‐1) and surface modified with Fas ligand (FasL). MCP‐1 was one of the key chemokines in inflammation that could regulate and mediate T cell migration to the site of inflammation, while FasL could bind with Fas receptor overexpressed on the membrane of activated T cells to trigger the programmed cell death. Under the dual action of MCP‐1 and FasL, apoptotic T cells released a large number of ApoBDs (**Figure** **10**A). After internalization by macrophages, ApoBDs could promote the transformation of inflammatory M1 macrophages into anti‐inflammatory M2 phenotype. Therefore, in this case, the immune tolerance microenvironment was reconstructed to ameliorate osteoporosis. In fact, the in situ release of ApoBDs in this work was not directly observed. Instead, the authors isolated ApoBDs derived from activated T cells under the treatment with nanoparticles in vitro and injected these generated ApoBDs into ovariectomized mice. It was found that the percentage of Th17 cells decreased while Tregs increased in ApoBDs‐treated mice, with blunted production of proinflammatory cytokines (Figure 10B). All these results suggested that T cell depletion nanoparticles may be through in situ production of ApoBDs, to ameliorate osteoporosis and rescue the osteogenic deficiency.

In the previously mentioned literature, ApoBDs were used to transfer genetic information, transduce signals between cells, or trigger signal transduction pathways. Recently, J. Sun's group^[^ ^72^ ^]^ reported that ApoBDs released from apoptotic cancer cells could deliver and transfer chemotherapeutics to neighboring tumor cells. When tumor cells underwent apoptosis under the action of chemotherapy drugs, the released ApoBDs could wrap and package cytoplasmic contents. Therefore, during this process, residual drugs in apoptotic cells could be stored in ApoBDs and be further internalized into an adjacent layer of cells via the “neighboring effect.”^[^ ^73^ ^]^ However, in the process of drug delivery by corresponding ApoBDs, drugs were gradually consumed. And limited to the hypoxia environment inside the tumor, the therapeutic effect was often not expected. In Sun's design, camptothecin (CPT) and hypoxia‐activated drug (PR104A)‐containing prodrug (CPT‐SS‐PR104A) was synthesized via a disulfide linkage and applied to self‐assemble into nanoparticles (Figure 10C). When nanoparticles were internalized into cells, CPT and PR104A could be released quickly under the high‐level cytosolic glutathione. First, external normoxic tumor cells underwent apoptosis under the action of CPT and drug‐containing ApoBDs were released. Then, these ApoBDs were engulfed by internal hypoxic tumor cells. Even though CPT was consumed by the external normoxic cells and the content of CPT in ApoBDs gradually decreased, PR104A was consumed less and could be activated in the internal hypoxic cells. Therefore, PR104A‐containing ApoBDs continued to exert the anti‐tumor effect in the core of the tumor. With the assistance of ApoBD‐mediated neighboring effect, they have proved that the assembled nanoparticles could achieve better penetration both in the in vitro 3D tumor spheroids (Figure 10D) and in vivo tumor spheroid penetration study (Figure 10E). In this study, ApoBDs were linked with intercellular drug delivery via neighboring effect. It was proved that apoptosis‐derived ApoBDs could wrap unacted drugs remained within cells and mediate the drug transfer between neighboring tumor cells, to enhance the penetration of drugs. Herein, apoptotic tumor cells themselves were used as the source of delivery medium to realize drug delivery and release. This study further enriched the means and mechanism of the organism's drug delivery.

## 6 Outer Membrane Vesicles

OMVs are naturally released by bacteria with bilayer spherical structures ranging in size from 20 to 500 nm.^[^ ^74^ ^]^ Under pathogenic conditions, bacteria can release OMVs to transfer bioinformation to other bacteria and improve their survival.^[^ ^75^ ^]^ As for commensal bacteria in humans, the released OMVs could elicit the immune system and showed obvious benefits to reinforcing the host defenses system. Nowadays, OMVs were widely applied in biomedical fields, such as vaccines to prevent infections, adjuvants or immunotherapy agents to treat cancer, drug delivery vesicles to target parent bacteria, and anti‐bacterial adhesion agents in chronic inflammatory diseases.^[^ ^76^ ^]^ Back in 1975, I. W. DeVoe and J. E. Gilchrist^[^ ^77^ ^]^ demonstrated that OMVs can be released in the cerebrospinal fluid from patients infected with meningococcus. Subsequently, more and more work identified the in vivo production and existence of OMVs by bacteria in infected tissues or gut.^[^ ^78^ ^]^

Although the application of OMVs in biomedical field is a research hotspot and the in vivo production of OMVs has been verified, few studies have taken advantage of the in situ release behavior of OMVs to treat diseases. Recently, a seminal study by G. Nie's group^[^ ^79^ ^]^ demonstrated that the in vivo OMVs generation propensity of bacteria can be used to develop novel bacteria‐derived oral cancer vaccine. As one of the most abundant symbiotic bacteria in the gut, *Escherichia coli* (*E. coli*) was engineered to express ClyA–Ag–mFc, of which ClyA protein was one of the most abundant proteins on the surface of OMVs, Ag was the tumor antigen, and mFc could facilitate the recognition and uptake of OMVs by DCs through the interaction between Fc and neonatal Fc receptor in DCs. When this genetically engineered *E. coli* were taken orally, they could colonize in the small intestine and secrete OMVs that were decorated with tumor antigens. The OMVs could pass the intestinal epithelial barrier, carry the cargo (antigens), and present them to the immune system together with the autologous adjuvants, thereby activating potent in vivo antitumor immune responses. In addition, to eliminate the immune tolerance caused by continual antigen stimulation, a “responsive switch,” arabinose (Ara), was added to trigger the OMVs release behavior of the engineered bacteria. Upon the administration of Ara, the retention of engineered bacteria in gut was enhanced. This strategy showed good efficacy in treating lung metastatic melanoma, subcutaneous colon cancer, and could induce long‐term immune memory. Compared with the group of engineered bacteria without Ara (ClyA–OVA–mFc(–Ara)), the engineered bacteria plus Ara (ClyA–OVA–mFc) exhibited significantly decreased number of lung metastases, suggesting the important role of “responsive switch.” This work could not only solve the epithelial barrier penetration difficulty of commensal bacteria, but also overcome the difficulties brought by complex digestive tract environment of conventional oral vaccines.

**Figure 11:**
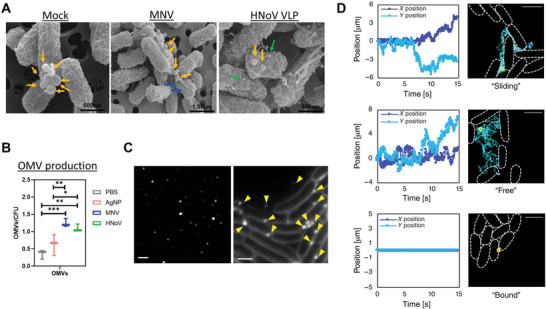
A) Scanning electron microscopy (SEM) images of *Enterobacter cloacae* with or without norovirus (MNV and HNoV VLP). The yellow arrow indicates OMVs and blue/green arrow indicates norovirus. B) The OMVs production amount when bacteria were treated with different groups. (**p < 0.01, ***p < 0.001). C) The Visualization of purified OMVs (left) and embedded *E. coli* producing OMVs (right) by fluorescence microscopy. D) OMVs trajectories by microscopy image and vesicle tracking analysis. A,B) Reproduced with permission.^[^ ^81^ ^]^ Copyright 2022, John Wiley & Sons, Inc. C,D) Reproduced with permission.^[^ ^82a^ ^]^ Copyright 2021, American Association for the Advancement of Science.

There are several ways to promote OMVs generation in vivo. This is important as it may reinforce the communication between vesicle‐releasing bacteria and the host, and thus enhance the therapeutic effect of disease. It was demonstrated that bacteria could respond to some stress conditions and enhance the release amount of OMVs, such as under the existence of antibiotics, virus infection, stressful environments, and other chemical agents.^[^ ^80^ ^]^ In 2021, C. A. Mosby and coworkers^[^ ^81^ ^]^ found that when a commensal bacterium, *Enterobacter cloacae*, were interacted with norovirus, the production amount of OMVs was enhanced, and particle size and accompanying bioinformation of OMVs were changed (**Figure** **11**A,B). The detailed mechanism was relevant to the altered genes related to membrane stability and OMVs production in bacteria. Through the isolation and purification of OMV from fecal pellets, it was found that the amount of OMV (MVs/mL) generated in norovirus‐infected mice was higher than that in mock‐infected mice. Since OMVs could easily pass through the intestinal epithelium, the authors speculated that the altered amount, size, and bioinformation of OMVs may facilitate the entry of virus into target cells, enhance the infectivity of virus, or modulate the body's immune response to infection. In another work, under the action of metronidazole in a subinhibitory concentration, the in vivo OMVs secretion amount of bacteria was also enhanced in Wistar rats infected with *B. fragilis*.^[^ ^80b^ ^]^ The in vivo release of OMV was verified by transmission electron microscope images in tissue cages model.

More importantly, the in vivo OMVs generation process can be real‐time monitored.^[^ ^82^ ^]^ In vivo monitoring of vesicle generation is helpful to elucidate the action mechanism and advantage of this in situ generation of biological vesicles strategy. J. Bos et al.^[^ ^82a^ ^]^ provided a novel method to monitor OMVs and overcome the visualization technical challenge of OMVs that were usually labeled with fluorescent lipophilic dye. The real‐time visualization of OMVs was achieved by using a high‐magnification wide‐field fluorescence microscopy and a scientific complementary metal‐oxide‐semiconductor (sCMOS) camera (Figure 11C). Their positions and trajectory were respectively recorded. The movement patterns of OMVs could be classified into the “sliding,” “free,” and “bound” state (Figure 11D). In addition, vesicle production amount and speed increased significantly when bacteria were attacked with low doses of antibiotics. Another work reported by M. Bittel^[^ ^82b^ ^]^ revealed that the OMVs medicated microbe‐host‐communication in vivo by administrating Cre‐recombinase expressing *E. coli* into *Rosa26.tdTomato* reporter mice. The biological information in bacteria can be transferred to the host intestinal stem cells and macrophages in intestinal epithelium. Distant tissue such as heart, liver, kidney, spleen, and brain, could also receive the bioinformation from bacteria.

## 7 Perspectives and Limitations

As a biological shuttle system, biological vesicles are the main means of cell–cell communication in the body, which can mediate the transmission of biological information between different cells, maintain the body's homeostasis, or regulate the immune response in the body. Promoting or decreasing the production of biological vesicles in organisms could be an intelligent strategy for the treatment of many diseases. In this review, we summarized the current approaches for disease treatment by a variety of in situ vesicles‐producing systems via living organisms, including direct delivery of engineered cells/bacteria to produce vesicles and inducing the generation of biological vesicles in vivo. Of course, given the heterogeneity and lack of control in vivo, it seems easier to determine the ideal vesicle composition and synthesize vesicles at high yields in vitro. However, endogenous‐cell‐generated vesicles may have more potential for development in terms of therapeutic effect, especially in the deep penetration of tumors. In tumor therapy, for example, exogenous vesicles need to target the focal tumor first. We know that intravenously injected vesicles are usually poorly targeted. Most of the endogenous‐cell‐generated vesicles depend on the tumor cells themselves to produce vesicles. The vesicles produced by tumor cells are more likely to be taken up by neighboring cells, performing communication functions, and even be taken up by other tumor cells as nutrients. Therefore, the in situ organism‐generated biological vesicles‐based strategy may have the potential to avoid a series of problems of conventional biological vesicles treatment, such as complicated purification steps, difficulties of in vivo long‐term circulation, inferior targeting ability of lesion sites, and the loss of some important bioinformatics molecules during the purification process. This strategy of in situ vesicles generation summarized here may change the delivery mode of vesicles from in vitro delivery to in situ generation of lesions, which may provide new inspirations and beyond‐expected effects for the treatment of diseases.

However, to date, this strategy is still in its infancy and belongs to a relatively novel concept. In the process of application, there are also many problems restricting its development:

*In vivo monitoring of the generation process of biological vesicles*: Biological vesicles are the intermediate between information exchange and material transfer. Monitoring the in vivo production process of biological vesicles and how biological vesicles convey bioinformation/drugs are of great significance to elucidate the mechanism of action of this drug delivery strategy in vivo. Due to the lack of reliable imaging and tracking tools, the in situ generation process of biological vesicles populations was often speculative, as these biological vesicles were hardly detectable or difficult to monitor. Most of the current methods to verify vesicle generation were indirect, including monitoring the in vitro generation process of vesicles by simulating in vivo conditions,^[^ ^43^, ^46^ ^]^ quantifying the number of in vivo generated vesicles,^[^ ^80^, ^81^ ^]^ and monitoring the penetration effect of drugs carried vesicles in tumor.^[^ ^55b^ ^]^ The failure of these methods to reflect the real‐time generation of vesicles and transportation of bioinformation/drugs in vivo may lead to many speculative conclusions about vesicle‐related biological mechanisms. At present, some researchers have realized in vivo monitoring of OMVs generation using advanced technologies,^[^ ^82^ ^]^ such as high‐magnification wide‐field fluorescence microscopy combined with sCMOS camera, and Cre‐LoxP system with Cre‐recombinase expressing *E. coli* and *Rosa26.tdTomato* reporter mice. These related techniques are worthy to be popularized in monitoring EV generation processes in other systems.

*Strategies for controlling and promoting biological vesicles generation in vivo*: Accurate release of vesicles at the lesion site and controlling the beginning and end of vesicle generation, are important means for the precise treatment of diseases. In fact, at present, many studies have attempted to achieve controlled release of vesicles. For example, PMPs were released when platelets came into contact with tumor cells,^[^ ^46^ ^]^ and OMVs were released by engineered bacteria only after Ara was taken orally.^[^ ^79^ ^]^ However, current studies mostly simply utilized the nature of spontaneous generation of biological vesicles by organisms. In this situation, precise treatment of diseases could only be enhanced by regulating the in vivo targeting properties of the biological vesicle‐producing organism or vesicle‐promoting nanodrugs. In addition, promoting the release of vesicle in vivo is another aspect of the dilemma. Promoting biological vesicles production in situ can help to reduce initial drug administration dose and improve the delivery efficiency of bioinformation or drugs, while few literatures have developed methods to enhance the generation level of vesicles in vivo. It is reported that cells, or symbiotic bacteria in the body, could produce more vesicles under stressed environment. Monensin could enhance the production number of vesicles. And when stimulated by norovirus, commensal bacteria would produce enhanced amount of OMVs.^[^ ^81^ ^]^ The method mentioned above to achieve accurate release of vesicles in the lesion site and promote vesicle production can be extended to other systems and disease treatment.

*Safety concern*: At present, vesicle‐based drug delivery systems are widely promoted and used because of their good in vivo biocompatibility. However, for the in situ organism‐generated biological vesicles, the in vivo safety evaluation will become more complex. First, since it is difficult to achieve fixed‐point or precise delivery of drugs to target cells, it is expected that other non‐target cells would generate heterogeneous vesicles. It is unclear how these heterogeneous vesicles will influence the safety and therapeutic effects of this endogenous vesicle generation strategy. Second, compared with conventional extracellular vesicles, the size and composition of such in situ generated vesicles will be more uneven and complex, and their purity will be hard to guarantee, leading to the difficulty in basic characteristics and quality control. As a result, systemic toxicity and immunogenicity become more difficult to figure out and control. In addition, whether there exists a risk of promoting tumor metastasis by promoting the production of tumor vesicles remains unknown. It was reported that tumor cells can release the exosome containing PD‐L1 to inhibit the in vivo antitumor response.^[^ ^83^ ^]^ And how to stop in situ tumor‐derived vesicle generation when the treatment is over is also a question to be considered.

*Clinical application*: The development of in situ‐generated vesicles is a relatively emerging field. Their clinical transformation potential is hard to evaluate. Currently, for these in situ organism‐generated biological vesicles, it is difficult to determine the effective number and purity of vesicles used in disease treatment. For extracellular vesicles produced in vitro, it requires several days and multiple large bioreactors to generate a sufficient dose of extracellular vesicles for treatment. It has been reported that depending on the route of administration, at least 3 million to 240 million MSCs were required to achieve a positive therapeutic effect in the preclinical studies of large animals (swine and sheep),^[^ ^84^ ^]^ which was equivalent to 1–100 of 150 cm^2^ cell culture flask.^[^ ^85^ ^]^ Although this in situ generation strategy can enhance the targeting of vesicles to some extent, the production and effective dosage of these in situ vesicles remain unclear and are still obstacles for clinical application. In addition, the transformation of this in situ vesicles generation delivery system is also affected by the clinical transformation of the administered vesicle‐promoting nanodrugs or vesicle‐generated cells. At present, the application prospects of cell therapy are relatively promising. As cell therapy progresses, the clinical application of cell‐based vesicle‐generation systems in vivo may be promoted. Moreover, in addition to using the basic characteristics of vesicle‐generated cells, it can also be engineered, such as surface modification or drug loading to produce functional or drug (chemical drug, dsRNA, or proteins)‐loaded vesicles.

*Limitations of cells and disease types*: Vesicles can be produced in a variety of cells, and the abnormalities in their production can lead to different diseases. Therefore, correcting the abnormal production of vesicles at the site of lesion by this in vivo vesicle‐generation strategy has a good application prospect in many diseases. At present, the parent cells of in vivo‐generated vesicles are mainly concentrated on tumor cells, platelets, or bacteria, and the types of related diseases are mostly limited to the tumor field. Expanding the application of this endogenous biological vesicles‐based strategy in other diseases may be a promising development direction in the future. In addition to bacteria, it has been reported that symbiotic fungi existed in vivo also have vesicle‐generating capacity. Promoting the generation of fungus‐derived vesicles in vivo may also have great research significance.

In conclusion, this method of in situ vesicle formation may provide a new paradigm for vesicle‐like drug delivery system in the treatment of diseases. With the development of novel technology, we believe that these limitations will be gradually overcome. This novel strategy will be widely applied in different diseases and promote the integration of different disciplines.

## Conflict of Interest

The authors declare no conflict of interest.
